# Proteasome inhibition, the pursuit of new cancer therapeutics, and the adaptor molecule p130Cas

**DOI:** 10.1186/1741-7007-9-72

**Published:** 2011-10-28

**Authors:** Dharminder Chauhan, Kenneth C Anderson

**Affiliations:** 1 The LeBow Institute for Myeloma Therapeutics and Jerome Lipper Myeloma Center, Department of Medical Oncology, Dana Farber Cancer Institute, Harvard Medical School, Boston, MA 02115, USA

## Abstract

Current interest in proteasome inhibitors for cancer therapy has stimulated considerable research efforts to identify the molecular pathway to their cytotoxicity with a view to identifying the mechanisms of sensitivity and resistance as well as informing the development of new drugs. Zhao and Vuori describe this month in *BMC Biology *experiments indicating a novel role of the adaptor protein p130Cas in sensitivity to apoptosis induced not only by proteasome inhibitors but also by the unrelated drug doxorubicin.

See research article: http:// http://www.biomedcentral.com/1741-7007/9/73

## 

The potential of proteasome inhibition in tumor therapy was first suggested by the success of the proteasome inhibitor bortezomib in the treatment of multiple myeloma, for which at the time of its approval in 2003 there was no effective therapy. Since then, the emergence of side effects and resistance [1] on the one hand, and the hope of developing the approach for other tumors on the other hand have led to extensive efforts to delineate the molecular mechanisms underlying the clinical effectiveness of proteasome inhibition with the aim of identifying new drugs acting on the same pathway. The discovery, now reported in *BMC Biology *by Zhao and Vuori, of an obligatory role for the focal adhesion protein p130Cas (Cas) in the cytotoxicity of bortezomib and a second proteasome inhibitor, MG132, illustrates some of the issues arising in connection with this quest.

## Pathways to destruction

**Figure 1:**
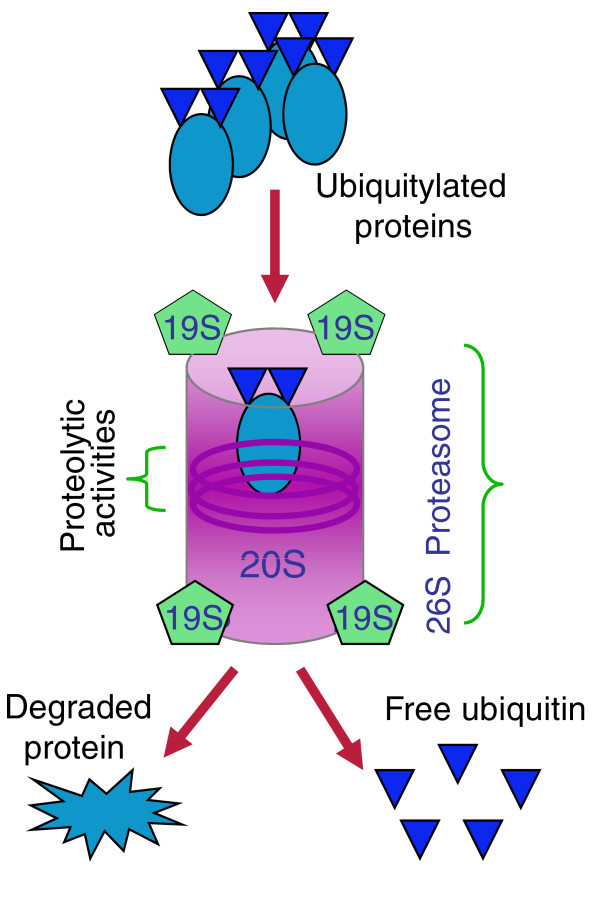
**Schematic representation of protein degradation by the proteasome**. Proteins are tagged for degradation by regulated ubiquitylation, which directs them to binding sites on the 19S regulatory subunits where they are unfolded for degradation in the barrel-shaped 20S catalytic core.

The ubiquitin proteasome system (UPS) plays an essential part both in the normal turnover of proteins and destruction of defective ones, and in the regulation of cellular proteins that maintain cell cycle progression, growth, and survival [2-5]. Proteins destined for degradation are tagged with ubiquitin and delivered to the proteasome, a large multi-subunit enzyme complex (Figure 1) whose barrel-shaped catalytic core contains three proteolytic activities - chymotrypsin-like (CT-L), trypsin-like (T-L) and caspase-like (C-L). Pharmacological inhibition of proteasome function results in intracellular aggregation of unwanted proteins, and this triggers cell death.

Although the proteasome is essential for the regulated degradation of proteins whose cyclic destruction is required for cell cycle progression, as well as of crucial cell signaling molecules, it is thought to be the accumulation of aggregated proteins that is responsible for the effectiveness of proteasome inhibition in the treatment of multiple myeloma. Multiple myeloma cells are derived from the antibody-producing cells of the immune system, and unlike other tumor cells, produce very large quantities of protein (the immunoglobulin chains that are their specialized product), which makes them unusually susceptible to the toxic consequences of inhibiting the normal degradative mechanisms. Normal cells can survive therapeutic doses of proteasome inhibitors because they have a lower rate of proliferation and consequently less need for proteasomal regulatory functions [3,4]. Moreover, inhibition of preoteasomal degradation upregulates autophagy [6], an alternative degradative pathway that delivers long-lived proteins, protein aggregates, and cytoplasmic organelles such as mitochondria to lysosomes for destruction [7]. Autophagy, which serves as an emergency source of energy during metabolic stress or starvation, can also contribute to the survival of tumor cells under stress [8]. Indeed, inhibition of autophagy enhances the induction of apoptosis by alkylating agents and irradiation in tumor cells, and can also synergize with bortezomib [9].

The studies of Zhao and Vuori [10] suggest that Cas may block this alternative pathway to survival in cells treated with proteasome inhibitors.

## Role of p130Cas in proteasome inhibitor-induced apoptosis

**Figure 2:**
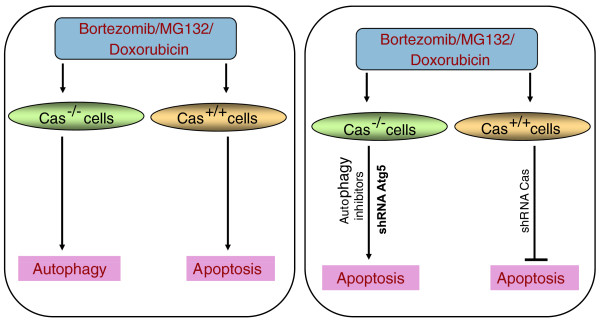
**Effects of bortezomib, MG132 or doxorubicin on mouse embryonic fibroblasts overexpressing Cas (Cas+/+) and Cas-deficient mouse embryonic fibroblasts (Cas-/-)**. shRNA, short hairpin RNA.

Cas is a docking protein that participates in the transduction of integrin- and/or cytokine receptor-induced growth and survival signaling [11], and is implicated in several pathological conditions, including inflammatory disorders, Alzheimer's disease, Parkinson's, developmental defects, as well as in cancer. Zhao and Vuori utilized both genetic and biochemical assays to show that Cas is required for proteasome inhibition-triggered apoptosis (Figure 2). Specifically, Cas-deficient mouse embroyonic fibroblasts (MEFs) were resistant to MG132- or bortezomib-induced cell death, while transfection with full length Cas (Cas-FL) restored the sensitivity of these cells to proteasome inhibitors. These data were corroborated with Cas small hairpin RNA-mediated knockdown experiments in other Cas-expressing cell types (human 293T and HeLa cells). This differential biological response in Cas-FL- and Cas-deficient cells to MG132 was not due to disparity in proteasome activity inhibition.

Several lines of evidence adduced by Zhao and Vuori [10] indicate that the effect of Cas on the apoptotic response to proteasome inhibitors involves blockade of autophagy. In MEFs lacking Cas, bortezomib triggers autophagy instead of apoptosis, but apoptosis is induced if autophagy inhibitors are added. Similarly, apoptosis is induced by proteasome inhibitors in cells lacking Cas if autophagy is prevented by downregulation of Atg5, a protein essential for formation of autophagosomes.

## Open questions

The mechanism whereby Cas mediates proteasome-inhibitor-induced apoptosis is not clear. One obvious possibility is that the proteasome inhibitors are acting by allowing the toxic accumulation of protein aggregates which would normally activate the autophagic pathway, and by preventing this, Cas is promoting the apoptotic response to the resulting stress. However, Zhao and Vuori found similar effects of Cas in response to the DNA damaging agent doxorubicin, suggesting that the role of Cas in response to apoptotic stimuli extends beyond that induced by proteasome inhibitors.

Earlier studies have shown that proteasome inhibition promotes Abl-mediated phosphorylation of Crk, disassembly of Cas-Crk complexes (cell migration/survival pathway), and cell death [12]. A role for c-Abl has also been reported in response to DNA-damaging agents such as ionizing radiation (IR) and alkylating agents such as cisplatin. Whether proteasome inhibition or doxorubicin affects c-Abl and Cas-Crk assembly resulting in cell death requires further examination. Similarly, the effect of proteasome inhibition on the role of Cas-associated proteins that are regulated by the ubiquitin ligase c-Cbl remains to be evaluated. The data showing that doxorubicin has the same effect as proteasome inhibitors in Cas-FL and Cas-deficient MEFs suggests that Cas can be regulated by mechanism(s) independent of proteasome inhibition as well. Nonetheless, the findings of Zhao and Vuori provide compelling evidence of a direct link between Cas and proteasome inhibition-triggered cytotoxicity.

Zhao and Vuori have been able to rule out one possible contribution of Cas to the apoptotic response to proteasome inhibition and doxorubicin. Cas has a known proapoptotic activity that has been attributed to its cleaved form (Cas-CT), and MG132 indeed induces Cas-CT. In contrast to earlier reports, however, this is not required for the induction of apoptosis since the overexpression of cleavage-resistant Cas mutants in Cas-deficient MEFs increases sensitivity to MG132.

The mechanism of the effects of Cas on the apoptotic response and on autophagy thus remains unclear, and the answer may require an understanding of the role of Cas in normal cells *in vivo*. Cas is an integral molecule linking integrin or extracellular matrix-mediated growth and survival signaling, and disruption of this adhesion-regulated pathway, or lack of Cas, may cause detachment-induced cell death (anoikis) and/or autophagy. Other focal adhesion proteins, such as paxillin, have been implicated in autophagosome formation; and Cas interacts with paxillin. This interplay between Cas and paxillin or other focal adhesion kinases (FAKs) may provide an additional link to autophagy.

In any case, it is clear that the specific conditions and cellular context will be important in the influence of Cas on tumor cell survival and drug sensitivity. A number of previous studies, as Zhao and Vuori acknowledge, have shown an association of aberrant expression of Cas with tumor cell survival and drug resistance. For example, the overexpression of Cas activates growth and survival signaling pathways via phosphoinositide 3-kinase/Akt, ERK1/2, epidermal growth factor receptor, Rac, or Src, conferring doxorubicin or tamoxifen resistance [11]; activation and aberrant expression of Cas correlates with tumor progression and metastasis; and overexpression is associated with poor prognosis and resistance to primary chemotherapeutic treatment in breast, lung, and prostate cancer, as well as glioblastoma and melanoma. This apparent conflict with the pro-apoptotic influence of Cas in the experiments reported by Zhao and Vuori may reflect aberrant effects of overexpression, or cell-type-specific effects, or both. Moreover, the observations of Zhao and Vuori are so far confined to mouse embryo fibroblasts, and will require further validation using primary cells from patients, as well as *in vivo *animal models, in order to assess clinical relevance. Quantitative assessment of Cas protein levels in patient tumor cells and their correlation with sensitivity to proteasome inhibitors may then provide further insight into selecting patient populations likely to respond. It is also worth pointing out that autophagy can help kill cells as well as rescue them [8].

Future studies in various cancer cell types examining Cas expression and associated responsiveness to proteasome inhibitors (and other drugs) may help to inform clinical trials of autophagy inducers or inhibitors in combination therapies.
